# Enhanced Resolution of Neutron Autoradiography with UV-C Sensitization to Study Boron Microdistribution in Animal Models

**DOI:** 10.3390/life13071578

**Published:** 2023-07-18

**Authors:** Agustina Mariana Portu, María Sol Espain, Silvia Inés Thorp, Verónica Andrea Trivillin, Paula Curotto, Andrea Monti Hughes, Emiliano César Cayetano Pozzi, Marcela Alejandra Garabalino, Mónica Alejandra Palmieri, Pablo Nicolás Granell, Federico Golmar, Amanda Elena Schwint, Gisela Saint Martin

**Affiliations:** 1 National Atomic Energy Commission (CNEA), San Martín C1429BNP, Argentina; msolespain@gmail.com (M.S.E.); silviathorp@cnea.gob.ar (S.I.T.); verotrivillin@gmail.com (V.A.T.); curotto@cae.cnea.gov.ar (P.C.); andre.mh@gmail.com (A.M.H.); eccpozzi@gmail.com (E.C.C.P.); marcegarabalino@gmail.com (M.A.G.);; 2 National Scientific and Technological Research Council (CONICET), Ciudad Autónoma de Buenos Aires C1425FQB, Argentina; fgolmar@unsam.edu.ar; 3 School of Science & Technology, National University of San Martín (UNSAM), San Martín B1650JKA, Argentina; 4 Department of Biodiversity and Experimental Biology, Faculty of Exact and Natural Sciences, University of Buenos Aires (UBA), Ciudad Autónoma de Buenos Aires C1428EGA, Argentina; monicaalepalmieri@gmail.com; 5 Micro and Nanotechnology Centre of the Bicentennial (CNMB), National Institute of Industrial Technology (INTI), San Martín B1650JKA, Argentina

**Keywords:** neutron autoradiography, nuclear track detectors, BNCT, boron microdistribution, animal models, UV-C, polycarbonate, PADC

## Abstract

The assessment of boron microdistribution is essential to evaluate the suitability of boron neutron capture therapy (BNCT) in different biological models. In our laboratory, we have reported a methodology to produce cell imprints on polycarbonate through UV-C sensitization. The aim of this work is to extend the technique to tissue samples in order to enhance spatial resolution. As tissue structure largely differs from cultured cells, several aspects must be considered. We studied the influence of the parameters involved in the imprint and nuclear track formation, such as neutron fluence, different NTDs, etching and UV-C exposure times, tissue absorbance, thickness, and staining, among others. Samples from different biological models of interest for BNCT were used, exhibiting homogeneous and heterogeneous histology and boron microdistribution. The optimal conditions will depend on the animal model under study and the resolution requirements. Both the imprint sharpness and the fading effect depend on tissue thickness. While 6 h of UV-C was necessary to yield an imprint in CR-39, only 5 min was enough to observe clear imprints on Lexan. The information related to microdistribution of boron obtained with neutron autoradiography is of great relevance when assessing new boron compounds and administration protocols and also contributes to the study of the radiobiology of BNCT.

## 1. Introduction

Cancer is one of the main causes of death worldwide, and for many of the types of this disease, the best treatment strategy is still a matter of research. Boron neutron capture therapy (BNCT) is a modality for cancer treatment based on the properties of high linear energy transfer (LET) radiation and the selective localization of a compound that transports the neutron capture element (^10^B). It requires the infusion of a ^10^B-enriched compound that selectively targets the tumor cells and the subsequent irradiation of the zone to be treated with a neutron flux of adequate energy. Thus, the BNC reaction (^10^B (n,α) ^7^Li) takes place and the unstable ^11^B atom disintegrates, emitting an alpha particle and a recoil ^7^Li ion in opposite directions. This way, localized damage is produced at the boron-loaded cell sites (e.g., [1]). Since its was proposed for the first time, BNCT has been applied to different malignancies with poor prognosis, such as glioblastoma multiforme, head and neck cancer, and melanoma, showing remarkable results [2,3,4,5]. Other targets that have been explored are osteosarcoma [6], lung [7] and liver [8] cancer, etc.

Currently, in a treatment instance, the estimation of the macroscopic concentration of boron in normal and tumor tissues is based on the concentration of boron measured in blood, assuming that the concentration ratio between tissues (i.e., the tumor/blood or normal tissue/blood) can be described as a unique value that remains constant throughout the whole procedure. This gross boron concentration is obtained by means of multielemental analysis techniques, such as inductively coupled plasma spectroscopy, both optical and mass emission (ICP-OES and ICP-MS), or analysis by neutron activation of instantaneous gamma radiation or prompt gamma, among others [9]. Although clinical dosimetry uses these macroscopic values to establish dose–response relationships, they have not always been explanatory of the observed response to therapy. Furthermore, every time a new target, strategy, or compound is to be assessed for the first time, radiobiological studies are essential to comprehend the mechanisms of BNCT for those particular tumors and their microenvironment and also to understand the therapeutic success probability [10]. In the context of the radiobiological studies, boron uptake by tumoral and normal tissue is a pivotal field of study.

Under these circumstances, the need to explore methodologies that address the microscopic scale then arises. It should be noted that knowing the exact localization of boron in the tumor and neighboring tissues represents a basic and necessary condition to adequately apply this type of therapy without causing damage to normal tissue. Moreover, knowledge of boron microdistribution at the cellular and tissue level is essential not only when considering a treatment but also for studying new boron compounds [11], and it is definitely fundamental when the suitability of BNCT in different biological models has to be evaluated [12,13]. Finally, the information extracted from the microdistribution analysis serves as an input for microdosimetry models [14]. Only a small number of techniques allow precise studies of boron microdistribution, such as secondary ion mass spectrocopy (SIMS, e.g., [15]) or laser-induced breakdown spectroscopy (LIBS) [16]. All of these techniques involve a high degree of complexity and sophistication.

In this context, neutron autoradiography is an attractive option due to its high resolution and low cost. This technique is based on the use of nuclear track detectors (NTD) and radiation-sensitive materials, either organic or inorganic. When a sample loaded with a particle-emitter element is put in contact with an NTD, the charged particles impact the detector and create zones of localized latent damage which can be enlarged up to light microscopy scale by a suitable chemical process. Thus, it is possible to produce an image from the observation in the detector of the tracks generated by the emitted particles [17]. Due to their versatility and high sensitivity, polymeric NTDs have been extensively used to generate autoradiographies of biological samples for different applications. For example, they have been applied to perform actinide bioimaging in tissues [18] and to determine uranium distribution in rat kidney sections [19]. In the context of BNCT, the autoradiographic image of a histological section coming from a boron-loaded sample is obtained by irradiating the sample-detector assembly with thermal neutrons and later etching to enlarge the particle damage. This way, an image is formed by the tracks of the particles emitted in the tissue [20,21].

In our laboratory, we have developed different approaches to neutron autoradiography. The co-registration of histological and autoradiographic images, where we observe differences in shades of gray, allows the determination of the boron atom distribution in the tissue sample (qualitative autoradiography) [22]. Moreover, these gray levels can be converted to preliminary boron concentration values through an optical density analysis [23]. In case events can be individualized, the concentration of ^10^B in different regions of a tissue section can be assessed by measuring the track density in the detector (quantitative autoradiography). Boron concentration values are correlated to nuclear track density through a calibration curve [24].

The autoradiography technique has been widely applied to address different radiobiological problems in the context of BNCT. The feasibility to treat limb osteosarcoma has been analyzed through the study of boron uptake by neoplastic and normal tissue, not only for BNCT mediated by BPA [13] but also for boric acid [25]. The extension of the neutron autoradiography to hard tissues was reported in [26]. Moreover, neutron autoradiography has also been implemented to evaluate the potential changes in boron microdistribution and its effects while incorporating new modalities to improve the efficacy of BNCT. For example, in [27], two methods whose therapeutic results had already been proven individually (blood vessel normalization prior to treatment and sequential BNCT) were combined, revealing an association between the boron microdistribution homogeneity in tumors and the achieved tumor control. Also, the improvement of the delivery of the boron compound sodium decahydrodecaborate (GB-10) by means of an electroporation treatment was evaluated through neutron autoradiography in a hamster cheek pouch oral cancer model [28]. In fact, a significant increase in the absolute and relative tumor boron concentration as well as an optimization of boron microdistribution were reported and suggested this strategy as a promising tool to improve the therapeutic efficacy of BNCT/GB-10. In [29] the feasibility of applying accelerator based BNCT for the treatment of osteosarcoma was explored, and measurements of boron concentration in tumor and surrounding tissues of interest were obtained by neutron autoradiography. Furthermore, in order to study the possibility of applying BNCT to treat diffuse pulmonary tumors, boron uptake in normal lung and in pulmonary metastases from rat colon adenocarcinoma were studied qualitatively [30] and quantitatively [31]. A uniform boron distribution in normal lung was found, and a selective ^10^B uptake in tumor tissues was proved.

For certain applications where the tissue regions to be evaluated are too small, the conventional quantitative autoradiography reaches its resolution limit. For example, in [32] we studied the boron accumulation of different compounds in the tissue structures of tumor and premalignant and normal cheek pouch in the hamster cheek pouch oral cancer model. When addressing the thin epithelium of premalignant tissue and normal cheek pouch, the co-registration of the histologic and autoradiographic images became challenging. It should be noted that the detector has to be separated from the sample prior to the etching process in order to assure a homogeneous chemical attack at the detector surface. The need to uncouple the biological sample from the detector may affect the spatial correlation between the emitter site (on an optical image of the tissue section histology) and the particle track location. This fact may affect the spatial resolution of the measurement and should be considered, depending on the requirements of the application and the kind of information to be obtained from the autoradiographic image.

These difficulties can be avoided by the simultaneous observation of both nuclear tracks and the sample image on the detector. In previous works, we presented a methodology to produce an “imprint” of cells cultivated on a polycarbonate detector by exposure of the detector to UV-C light (254 nm wavelength) [33]. A mechanism of photodegradation occurs on the surface of a polycarbonate foil when it is exposed to UV-C to a degree that depends essentially on the irradiation time. As a consequence, the bulk velocity (Vb, velocity at which the etching solution attacks the undamaged material) increases significantly. So if an assembly biological sample-NTD is exposed to UV-C light, the radiation effect on the detector is not uniform, as the heterogeneities of the different structures of the sample mask incident light in a differentiated way. Later the etching solution will attack at different velocities throughout the detector surface and an “imprint” of the biological material will be formed. If sample structures and nuclear tracks are superimposed, a more precise knowledge of the localization of boron atoms can be achieved. The advantage of this approach is that both the imprint and the tracks are revealed in the same process. We have applied this technique for the analysis of melanoma cell lines [34] and optimized it for breast cancer cells [35].

Tissue structure largely differs from that of cultured cells since it contains multiple cell types and extracellular matrix components. Sections obtained by microtome preserve the architecture and spatial organization of the tissue. This way, several aspects must be considered in order to extend the methodology developed for cell cultures to tissue sections. Thus, the aim of this work is to present an extension of the UV-C autoradiography technique for the analysis of boron microdistribution in tissue sections. We studied polycarbonate (PC) as the NTD for that purpose and optimized the experimental conditions to obtain good quality tissue imprints. We also evaluated the ability of polyallyldiglycolcarbonate (PADC) to produce imprints. A variety of tissues of interest were assayed when enhancing neutron autoradiography resolution, and the workflow involved in the generation of images of nuclear tracks and tissue imprints was fully revisited.

## 2. Materials and Methods

### 2.1. Biological Samples

Different animal tissues were used to test the methodology leading to the UV-C imprint formation. The biological samples used in this study came from experimental protocols approved by the Argentine National Atomic Energy Commission Animal Care and Use Committee (CICUAL-CNEA). No animals were specifically sacrificed to perform this work.

- Samples coming from normal BDIX rats (Charles River Lab., Wilmington, DE, USA). The animals were housed as previously described (e.g., [36]) Reference organs (liver, kidney, and lung) of normal rats excised 3–4 h post intravenous infusion of BPA (18 mg ^10^B kg^−1^ body weight (bw)) or GB-10 (50 mg ^10^B kg^−1^ bw) [37,38].
- Samples coming from the hamster cheek pouch oral cancer model: tumor and premalignant tissue samples were excised after 3 h intravenous infusion of BPA (15.5 mg ^10^B kg^−1^ bw, as reported in [39,40]).

After extraction, tissue blocks were preserved in liquid nitrogen to avoid boron migration and then sectioned at 5 μm, 10 μm, and 30 μm using a cryostatic microtome (CM 1850, Leica Microsystems, Heidelberg, Germany). The sections were immediately mounted on NTD sheets to perform neutron autoradiography. In addition, other tissue samples were mounted in quartz slides for the registration of UV-Visible absorption spectra on a GBC UV/VIS 920 spectrophotometer in the wavelength range from 200 to 856 nm.

### 2.2. Nuclear Track Detectors

Polycarbonate (Lexan^TM^) and polyallyldiglicol carbonate (CR-39) films of 250 μm and 1 mm thickness, respectively, were tested as NTDs to evaluate the imprint formation. In our laboratory, the development of the different approaches to neutron autoradiography is based on the use of Lexan sheets as NTD; since it is an easy-to-manipulate material, it does not require a long etching time to reveal the nuclear tracks, and, mainly, it is not sensitive to the protons emitted in the neutron capture reaction of ^14^N (NNC), a highly present element in biological tissues [41]. The thresholds of different types of track detectors have been widely studied elsewhere [42]. Briefly, the formation of etchable tracks depends on the number of primary ionizations produced along the trajectory of the particles. The number required for Lexan is higher than the ionization produced by proton particles along all the energy/nucleon range. However, the etching conditions can be adapted in order to etch almost every type of particle that could impact CR-39. In particular, the peak energy of protons emitted in the NNC is around 584 keV and they can be observed on CR-39 after an etching with NaOH [43].

**Figure 1:**
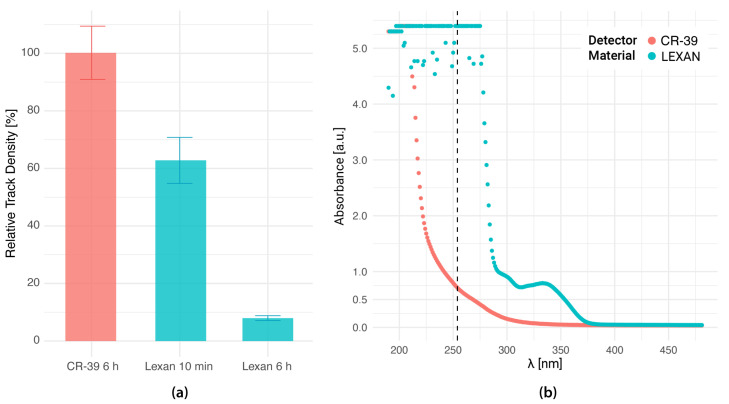
The response of CR-39 and Lexan to UV-C. (**a**) Relative track density for BNC alpha and Li tracks in CR-39 after 6 h UV exposure and in Lexan after 6 h and 10 min UV exposure (λ = 254 nm). Etching: KOH solution at 70 ${}^{\circ }$C for 2 min (Lexan) NaOH solution at 70 ${}^{\circ }$C for 22 min 35 s (CR-39). 50 images were analyzed for each condition, including the reference samples without UV irradiation. Results are reported as the mean value ± 1 SE. (**b**) UV–visible absorbance spectrum of virgin CR-39 and Lexan films. The dotted line shows the wavelength at which the samples were irradiated for the biological imprint generation (λ = 254 nm).

Moreover, the methodology to produce the imprint of cell cultures by UV-C sensitization has been developed using Lexan PC as NTD [34,35]. Nevertheless, we have reported a track-fading effect in PC that implies a decrease in the number of alpha and Li ion tracks observed in polycarbonate foils exposed to UV-C light [44]. The fading effect has been evaluated by means of the relative track density (RTD):

(1) RTD(thUV)=Trackdensity(thUV)/Trackdensity(0hUV),

where 0hUV corresponds to detectors that were not exposed to UV-C and thUV corresponds to foils exposed during t hours to ultraviolet light. In Figure 1a, the RTD for BNC alpha and Li tracks in Lexan and CR-39 detectors generated from agarose gels prepared with boric acid solutions is shown for different UV-C (λ = 254 nm) exposure times. The impact of UV-C sensitization appears to be negligible on CR-39. On the other hand, the relative track density in Lexan foils is 60% when irradiating for 10 min and dramatically decreases up to 10% for a 6 h UV-C exposure, as previously reported [44]. To analyze this behavior, the UV-visible absorption spectrum was measured in both detector materials. The results are illustrated in Figure 1b, where it is observed that absorption curves are different for each material. Moreover, for λ = 254 nm, Lexan absorbance is more than 10 times higher than that of CR-39. This fact could explain the different response to UV-C of these two polymers.

### 2.3. Nuclear Tracks Generation: Neutron Irradiation

The “NTD+tissue section” assemblies were irradiated with a neutron flux at the thermal column facility of the RA-3 nuclear reactor (Ezeiza Atomic Center, Buenos Aires) under established conditions that ensure uniformity and a correct determination of the total flux [45]. Neutron fluences of 10^12^ n cm^−2^ and 10^13^ n cm^−2^ were employed [24]. To verify stability, the neutron flux was monitored at specific reference points during the irradiations by means of an SPND (Self-Powered Neutron Detector).

### 2.4. Imprint Generation: UV-C Exposure

As stated above, to obtain the biological imprint on the detector film it is necessary to interpose an UV-C light sensitization step before removing the tissue from the detector. An irradiation device previously described was employed to expose the assemblies to UV-C light [33]. It contains a 15 W UV lamp with 254 nm wavelength emission (G15T8, Philips, Amsterdam, Holland). Prior to irradiation, the homogeneity of the field was tested using a radiometer (International Light Technologies ILT77). Preliminary studies showed that 10 min of UV-C sensitization of PC resulted in clear biological prints without excessive fading effect. However, other exposure times were also assayed, ranging from 5 min to 6 h. Typically, samples were exposed to UV-C after neutron irradiation, unless specifically stated.

### 2.5. Histological Analysis

The histological samples were explored before being removed from the detector. As both Lexan and CR-39 are transparent materials, the observation process can be performed on the tissue section adhered to the detector [22]. For this purpose, hematoxylin and eosin (H&E) stainings were applied. Characteristic staining times were chosen: 15 min for H and 3 min for E. The exploration of regions of interest (ROIs) was performed with an optical microscope (Olympus BX51) coupled to a CCD camera (Olympus DP70) [33]. The ROIs were photographed at increasing magnification, ranging from 1.25x up to 40x.

In order to examine the tissue surface, some samples were metallized with gold and observed in a scanning electron microscope (SEM) (FEI QUANTA 200), as reported in [34].

### 2.6. Imprint and Track Etching

Prior to the chemical attack of the NTD, the biological material was removed from the detector surface with trypsin-EDTA. The etching of both nuclear tracks and imprints was performed with the corresponding solution, depending on the polymer. For Lexan, a PEW (30 g KOH + 80 g ethyl alcohol + 90 g distilled water) solution at 70 ${}^{\circ }$C was used for 2 min and 4 min. For CR-39, a chemical attack with 6.25 N NaOH solution at 70 ${}^{\circ }$C was carried out for 22 min 35 s and 32 min 35 s.

### 2.7. Observation and Analysis

After the chemical attack, the detector foils were re-explored and photographed to look for the previously delimited ROIs during the histological analysis. The imprint and nuclear track images could be observed in the same field by slightly modifying the focus. To count nuclear tracks individually, images were captured throughout the entire sample with a Carl Zeiss MPM 800 microscope at 40x magnification. A threshold segmentation of tracks was performed with Image Pro Premier^TM^ software, allowing its subsequent quantification. In order to obtain boron concentration values from nuclear track density, the calibration curve previously constructed with boron standard solutions was applied [31]:

(2) $$\mathrm{track}\mathrm{density}=\left(3.40\;\;\pm \;\;0.09\times 10^{-4}\right)*{[}^{10}B]+\left(1\;\;\pm \;\;1\times 10^{-3}\right)\;R^{2}=0.99.$$

## 3. Results and Discussion

### 3.1. Observation of Imprints and Nuclear Tracks

**Figure 2:**
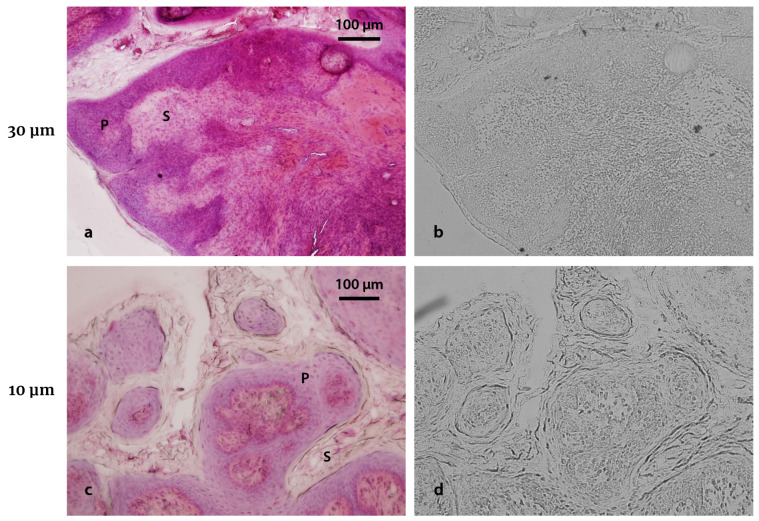
Imprint observation in tissue sections. (**a**) Tumor sample from the hamster cheek pouch oral cancer model (30 μm thickness section; 3 h post BPA infusion, 15.5 mg ^10^B kg^−1^ bw) stained with H&E and (**b**) its corresponding imprint in Lexan. (**c**) Tumor section (10 μm thickness; 3 h post BPA infusion, 15.5 mg ^10^B kg^−1^ bw) stained with H&E and (**d**) its corresponding imprint. Parenchyma (P) and stroma (S) regions are marked in both histological images. UV-C exposure time: 10 min. Etching time: 4 min. Original magnification: 20x.

The absorbance of UV light by the biological material may vary according to the different histological structures. When the tissue is put in contact with an NTD and exposed to UV-C, the photodegradation will not be uniform in the detector’s surface. After the etching process, an imprint that reproduces the tissue topography will be revealed. The correlation between the histological and imprint images is illustrated in the example of Figure 2, corresponding to a tumor from the hamster cheek pouch oral cancer model. The contours observed by optical microscope clearly reflect the histological characteristics of the sample, reaching cellular resolution specially in thin samples. The parenchyma and stroma regions observed in the histology (Figure 2c) are well defined in the imprint corresponding to the 10 μm thickness sample (Figure 2d). On the contrary, Figure 2b shows a more diffuse imprint of Figure 2a: the internal contours of the structures are not so well defined in the imprint corresponding to 30 μm, although it does allow us to clearly identify the edge of the sample on the detector (Figure 2b, bottom left). Both the tissue imprint and the nuclear tracks forming the autoradiographic image are chemically etched at the same time and can be observed by modifying the focus in the region of interest. This first observation confirms the feasibility of extending the UV-C imprint technique developed for in vitro samples to tissue sections. However, it also exhibits the need to revisit the workflow in order to obtain sharp and informative imprints.

**Figure 3:**
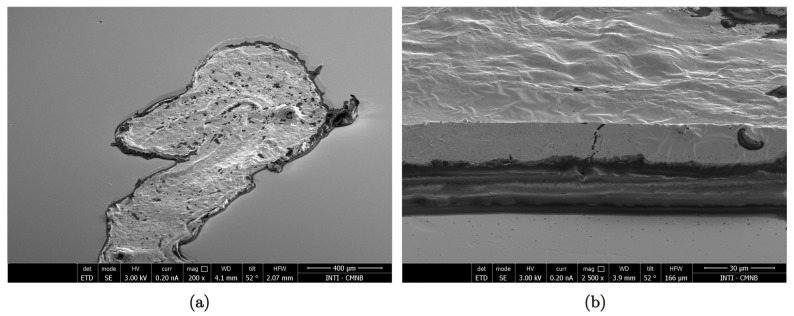
Scanning electron microscopy (SEM) images of a tissue section. Top view 200x, (**a**) and corresponding tilted view 2500x, (**b**) SEM of a normal cheek pouch section (30 μm thickness). The images were obtained with a focused ion beam/SEM (Dual Beam FEI Helios Nanolab 650), and the sample preparation was previously described [34].

Not only the slicing thickness will determine the imprint characteristics. The complexity of the imprint image stems from the irregular surface of the tissue samples. To illustrate this fact, an example of an SEM image of a tissue section belonging to a normal hamster cheek pouch is shown in Figure 3. The roughness observed in the top view at lower magnification, which translates into different brightness levels, is better observed in the tilted view. Although the texture of the surface is thin in comparison to the tissue thickness, the roughness implies differences in UV-C absorbance that are imprinted on the NTD with high definition. It should be noted that the nominal slicing thickness of the illustrative sample is 30 μm, but the final thickness is lower. This fact can be explained due to the evaporation of tissue water content that occurs after freeze-sectioning [46]. This issue will be further discussed in Section 3.7.

### 3.2. Neutron Fluence

**Figure 4:**
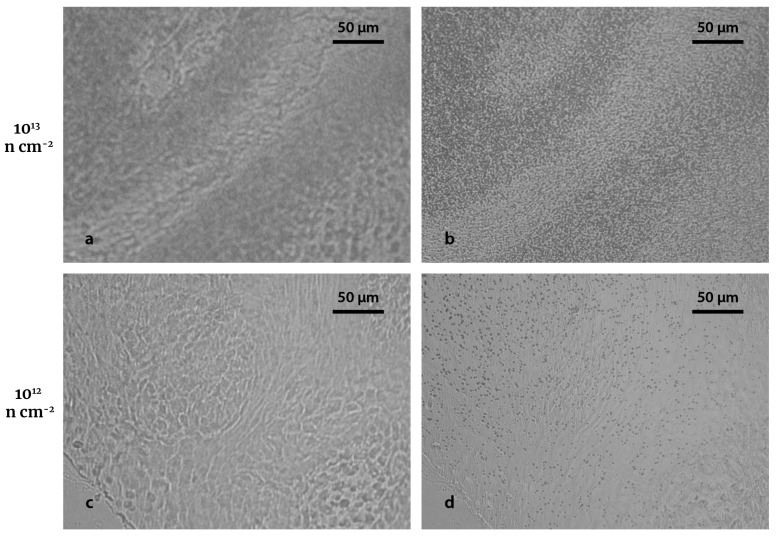
Imprints and nuclear tracks for different fluences. (**a**,**b**) are the biological imprint and the nuclear tracks, respectively, of a hamster cheek pouch tumor section (30 μm thickness; 3 h post BPA infusion, 15.5 mg ^10^B kg^−1^ bw) after a neutron fluence irradiation of 10^13^ n cm^−2^. (**c**,**d**) are the biological imprint and the nuclear tracks, respectively, of a hamster cheek pouch tumor section (30 μm thickness; 3 h post BPA infusion, 15.5 mg ^10^B kg^−1^ bw) after a neutron fluence irradiation of 10^12^ n cm^−2^. Both autoradiographic images were exposed to 10 min UV-C radiation and 4 min etching. Original magnification: 40x.

The fact of having both images superimposed represents an advantage in terms of the spatial correlation between tissue contours and BNC sites. However, the track observation and subsequent counting procedure is more complicated than in conventional autoradiography. Figure 4 shows the imprint (left) and the tracks (right) of autoradiographs from hamster tumor samples infused with BPA and irradiated with two different neutron fluences: 10^13^ n cm^−2^ (a,b), and 10^12^ n cm^−2^ (c,d). A fluence of 10^12^ leads to a low track density, thus preventing track overlapping, which allows individual visualization and quantification, as can be seen in Figure 4d. On the other hand, a 10^13^ n cm^−2^ fluence results in a high track density and the overlapping of the tracks generates a collective effect that allows studying the boron uptake by means of the grayscale (Figure 4b). Comparing the resulting images, a neutron fluence of 10^12^ n cm^−2^ seems to be more suitable for this novel approach, since tracks are sufficiently spread out to observe a clear correlation between the imprints and the nuclear tracks. A higher fluence hinders the identification of the imprint and the structure’s outline (Figure 4a).

The regions exhibiting a higher track density correspond to an increased boron uptake. As previously reported, distribution of BPA in tumor is not homogeneous as it is preferentially accumulated in parenchyma [32]. Further work will be devoted to apply this technique to premalignant and normal cheek pouch, where (as already mentioned in the Introduction section) the thin epithelium could be better delimited by the contours created after UV-C exposure.

Based on the observations of this section, neutron fluence used from now on will be 10^12^ n cm^−2^.

### 3.3. Evaluation of Homogeneous Samples

**Figure 5:**
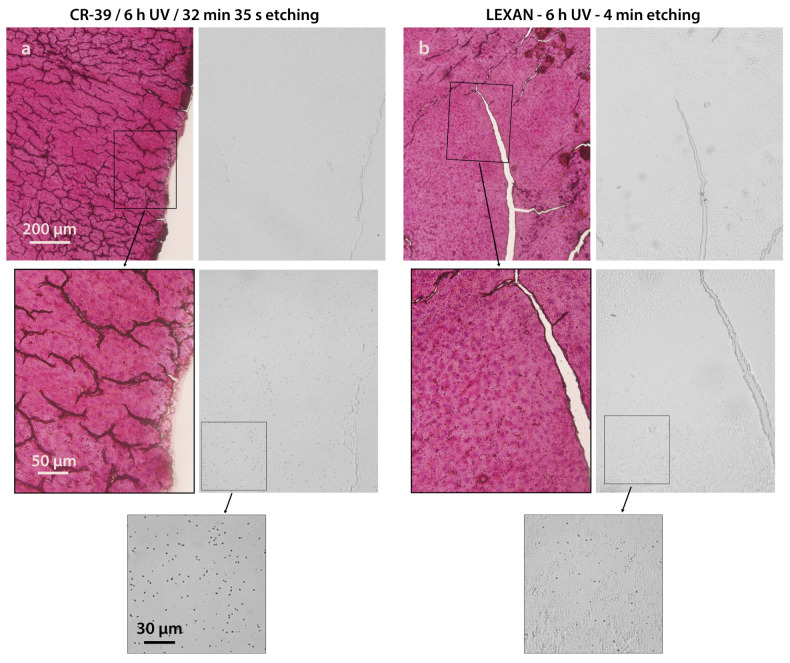
Tissue imprints of liver on different NTDs. Histological images sections sliced at 30 μm thickness and their corresponding imprint in CR-39 (**a**) and Lexan (**b**). Boron biodistribution: 4 h post BPA infusion, 18 mg ^10^B kg^−1^ bw. Neutron fluence: 10^12^ n cm^−2^. UV-C exposure time: 6 h. Etching time: 32 min 35 s for CR-39 (**a**), 4 min for Lexan (**b**). Original magnifications: 10x (**upper** panel), 40x (**central** panel), and 100x (**lower** panel).

Liver tissue was chosen for first assessments due to its uniformity, both at the histological level and in terms of boron distribution [47]. Figure 5 shows two BDIX rat liver sections (30 μm of nominal thickness) coming from the same sample and their corresponding autoradiographic images using CR-39 (Figure 5a) and Lexan (Figure 5b) as NTD, photographed at low and high magnification. A neutron fluence of 10^12^ n cm^−2^ and 6 h of UV-C exposure were the chosen conditions, based on our previous experience. The etching times were 32 min 35 s for CR-39 and 4 min for Lexan. In both cases, the biological imprint achieved under these conditions can be identified, as well as tracks generated by alpha and Li particles from the BNC reaction, mostly in images captured at higher magnification. Nevertheless, it should be noted that the imprint in CR-39 is not clear, allowing us to identify the boundaries and scratches but not the irregularities in the section surface. Moreover, at lower magnification (1.25x, not shown), it is not possible to distinguish the biological imprint in CR-39. Shorter UV-C exposure times produce no observable imprints (not shown). Conversely, the imprint on Lexan allows for a more detailed visualization of the tissue surface in addition to distinguishing boundaries and scratches. On the other hand, the autoradiography generated on Lexan exhibits fewer tracks than in CR-39. This fact points to the severe fading effect on Lexan caused by a 6 h exposure to UV-C light [44]. In order to avoid such a severe fading effect in Lexan, considerably shorter UV-C irradiation times were evaluated. Before moving on to the fading analysis, the determination of the etching time for Lexan is presented.

### 3.4. Tuning the Conditions for Lexan

The above results indicate that long exposures of PC detectors to UV-C are not convenient in order to determine realistic values of track density. It has been established elsewhere [44] that the photodegradation mechanism interferes with the revealing process of latent tracks (increase in the etching velocity), producing a considerable fading effect. However, for a 10 min sensitization period, the effect is not that severe and could be rectified using a correction factor. In addition, a short irradiation time allows speeding up the autoradiographic image generation process, which is also desirable.

**Figure 6:**
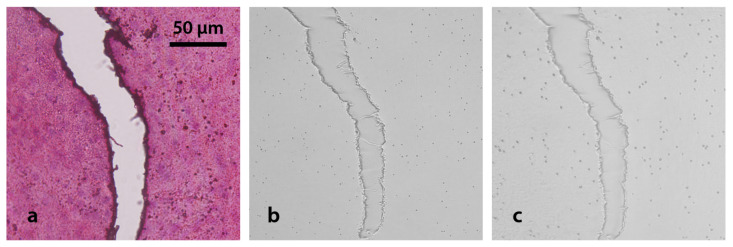
Evaluation of etching time in Lexan. (**a**) Liver tissue section (30 μm; 4 h post BPA infusion, 18 mg ^10^B kg^−1^ bw) and (**b**,**c**) their corresponding autoradiographic images in Lexan after 2 min and 4 min of etching, respectively. Neutron fluence: 10^12^ n cm^−2^. UV-C exposure time: 10 min. Original magnification: 40x.

Figure 6 shows a histological liver section and the corresponding autoradiographs generated with a neutron fluence of 10^12^ n cm^−2^ and a UV-C exposure of 10 min. The same sample was first etched for 2 min (Figure 6b) and an additional 2 min was later applied (total etching time: 4 min) for comparison purposes (Figure 6c). Under both conditions (2 and 4 min etching time), the contour of the scratches and the nuclear tracks are visualized, while an increase in track sizes is observed for a longer etching time, as expected. Thus, the condition “10 min of UV-C exposure + 4 min of UV-C” produces clear images that accurately reproduce the histological structures, as illustrated in Figure 2. Unless specifically stated, these will be the conditions applied for samples mounted on Lexan from now on.

It should be noted that, the larger the nuclear tracks, the higher the probability of overlapping. This fact should be taken into account in samples with high boron concentrations. The track density observed in Figure 2 is within the range of values typically observed in our experiments. In case of track overlapping, shorter etching times or smaller fluences should be applied.

### 3.5. Fading Effect

**Figure 7:**
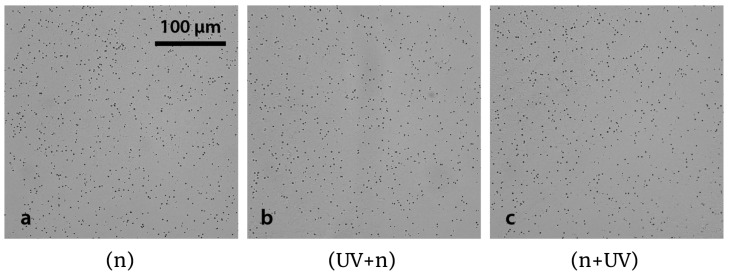
Evaluation of the sequence UV-C and neutron irradiation. Nuclear tracks in Lexan foils coming from sections of normal BDIX rat liver (30 μm thickness; 3 h post BPA infusion, 15.5 mg ^10^B kg^−1^ bw). The samples were treated with three different conditions: (**a**) no UV-C exposure (n), (**b**) exposed to UV-C before neutron irradiation (UV+n), and (**c**) exposed to UV-C after irradiation (n+UV). Neutron fluence: 10^12^ n cm^−2^. UV-C exposure time: 10 min. 4 min etching. Original magnification: 40x.

In order to evaluate the fading effect on Lexan due to UV-C exposure when interposing a tissue section, track density was studied in irradiated consecutive samples of 30 μm thickness liver sections under three different conditions: neutron irradiation only (n), exposure to UV-C light, and subsequent irradiation with neutrons (UV + n) and vice versa (n + UV). Examples of the resulting autoradiographic images are presented in Figure 7, where no histological imprints are visualized since photographs were captured in tissue uniform regions, without scratches. It can be observed that nuclear tracks look similar, regardless of the presence and order between the UV-C sensitization and the neutron irradiation. As expected, the event distribution seems to be uniform, thus confirming the homogeneous accumulation of BPA in normal liver.

Relative track density (RTD) was calculated for the three mentioned irradiation conditions (Table 1). The results show that samples exposed to UV-C have a lower mean value with respect to the (n) condition, although no statistically significant difference was found in RTD. The order between the UV-C and neutron irradiation does not affect the track density, as previously reported for nude detectors (with no tissue sample attached) [44]. It must be noted that the neutron irradiation is a critical step for the neutron autoradiography technique. Before the irradiation, potential contamination of the sample could imply the increase of nuclear tracks not related to the boron concentration of the sample. For that reason, the samples are handled with extreme care to avoid contamination. Thus, right after obtaining the sections they are mounted on the irradiation holder. After the irradiation, the handling of the samples becomes less critical (the latent tracks are already formed). This way, the UV-C exposure will be perfomed after the neutron irradiation. In summary, the order n+UV is more convenient in terms of workflow and to ensure adequate results.

In order to evaluate the impact of UV-C exposure on the boron microdistribution analysis, track density measurements for each condition were converted to ^10^B concentration values. As expected, the results are equivalent for the three conditions and also consistent with previously reported values [38]. This fact could be explained by the UV-C absorbance of the tissue section, that would act as a protection for the detector. This way it would prevent the fading effect from occurring.

### 3.6. Absorbance of Tissue at Different Thicknesses

**Figure 8:**
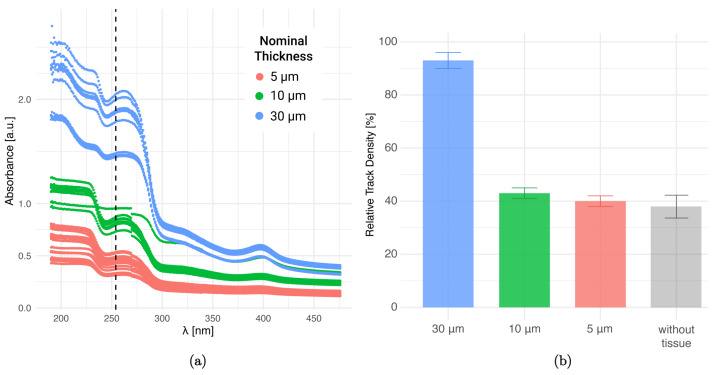
Effect of tissue thickness on absorbance and track density. (**a**) UV–visible absorbance spectrum of BDIX rat liver sections of different thicknesses. The dotted line shows the wavelength at which the samples were irradiated for the biological imprint generation (λ = 254 nm). (**b**) Relative track density for autoradiographic images in Lexan generated from liver sections of different thicknesses irradiated with 10^12^ n cm^−2^, exposed to 10 min UV-C (λ = 254 nm) and revealed with PEW for 4 min.

The absorbance of liver tissue sections was measured in order to analyze how this “shielding effect” might vary as a function of tissue thickness. As shown in Figure 8a the different spectra follow the same general behavior, although an offset is observed between the three groups: the absorbance increases with the sample thickness. The differences between measurements corresponding to samples of the same thickness could be ascribed to the shape irregularities of the sections. The range corresponding to UV-C radiation (100–280 nm) is the one with the highest absorption for all cases, and a considerable drop is found for wavelengths higher than 280 nm. This low absorption at larger wavelengths is consistent with the absence of imprints in samples exposed to UV-A radiation (300–400 nm), that was reported in a previous work [34]. For the wavelength of interest (around 254 nm), absorption differences are observed, mainly between the 30 μm sections and those of 5 μm and 10 μm thickness.

In Figure 8b the relative track density values for liver samples after a 10 min UV-C exposure are presented for different thicknesses. For 5 μm and 10 μm sections, the RTD is significantly lower than for the 30 μm samples (less than 50%), thus exhibiting a considerable fading effect for thinner samples. This correlates with the previously observed behavior of the absorbance curves at λ = 254 nm. The equivalence between samples of 5 μm and 10 μm could be ascribed to uncertainties in the thickness nominal value when sectioning with a cryostatic microtome. An extra value for the “without tissue” condition is presented. This result (gray bar) corresponds to the irradiation with neutrons of the “tissue+NTD” and the subsequent removal of the tissue section with trypsin prior to UV-C exposure. The RTD value seems to be equivalent to those of the 5 μm and 10 μm condition.

It should be noted that RTD was chosen for comparison purposes instead of absolute nuclear track density values, since the amount of nuclear tracks increases with thickness up to a plateau value [31]. In summary, the fading effect is more severe for thinner samples, as expected. This fact must be taken into account to correct the final boron concentration value.

### 3.7. Analysis of Heterogeneous Samples

**Figure 9:**
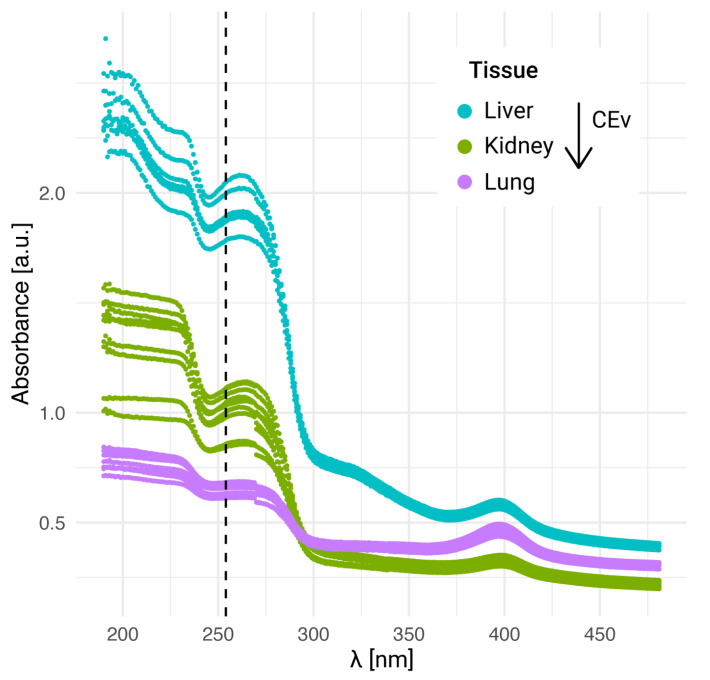
UV–visible absorbance for different tissues BDIX rat tissues. Nominal thickness sections: 30 μm. The dotted line shows the wavelength at which the samples were irradiated for the biological imprint generation (λ = 254 nm).

The extension of the UV-C autoradiography technique to tissue sections is of particular interest when analyzing heterogeneous tissues, especially in those scenarios where the structures to be studied reach the resolution of the conventional autoradiography technique. With this purpose, reference tissues with a non-uniform histology were chosen to further evaluate the imprint formation on polycarbonate. Firstly, UV absorbance spectra of lung and kidney tissue Sections (30 μm of nominal thickness) were studied and compared to those obtained for liver. Figure 9 shows the measurements corresponding to the same working day. In spite of potential differences within the samples due to non-uniformities, the spectra obtained for each tissue are reproducible. All the curves follow the same behavior as previously described in Figure 8, although an offset is observed for the different tissues.

Since samples are obtained by freeze-sectioning, when mounted on the NTD, they reach room temperature. Thereby, they undergo the evaporation of their original water content, which leads to mass loss and to a reduction in section thickness (nominal thickness). This phenomenon can be characterized by an evaporation coefficient (CEv), defined as the mass ratio between the dried and the wet sample [48]. The CEv value also serves to correct the boron concentration value obtained by nuclear track quantification. The reported BDIX rat tissue CEv values for BDIX liver, kidney, and lung are 0.30 ± 0.02, 0.23 ± 0.02 and 0.20 ± 0.02 respectively [46]. In Figure 9 all the analyzed sections were sliced at the same nominal thickness, but the final thickness can be estimated using the CEv of each tissue as a correction factor, obtaining values of 9 μm, 6.9 μm, and 6 μm for liver, kidney, and lung, respectively. Therefore, the differences in final thickness that the tissue sections actually reach cannot be discarded as one of the reasons to explain the observed offset in their absorbance profiles.

It must be noted that in this work we have not analyzed variations in tissue composition that could potentially imply a differential absorption profile. If the proposed technique was applied for a quantitative microdistribution study, it would be necessary to define the optimal thickness depending on the specific tissue to be analyzed along with a deeper understanding of the characteristic absorbance profile.

### 3.8. Imprint Formation at Different Tissue Thicknesses

**Figure 10:**
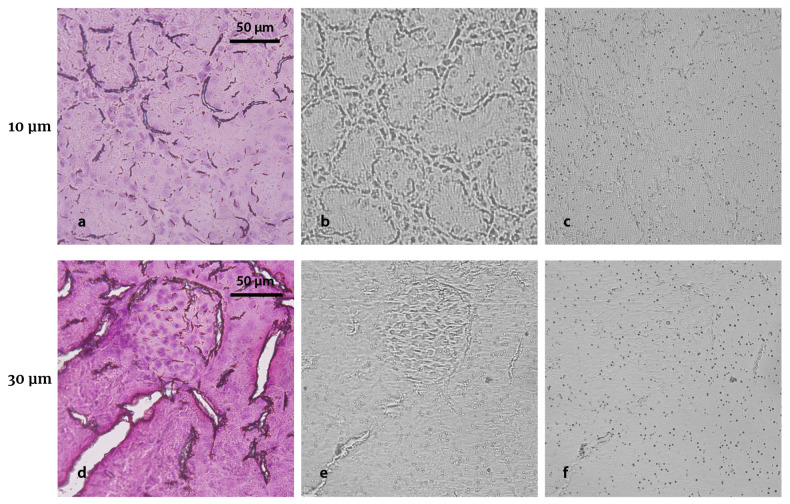
Effect of tissue thickness on imprints and tracks in heterogeneous samples. Normal BDIX rat kidney sections (4 h post BPA infusion, 18 mg ^10^B kg^−1^ bw) sliced at 10 μm (**a**) and 30 μm (**d**) nominal thickness and stained with H&E. The corresponding imprints in Lexan (**b,e**) and nuclear tracks (**c,f**) are shown. Neutron fluence: 10^12^ n cm^−2^. UV-C exposure: 10 min. 4 min etching. Original magnification: 40x.

In a previous approach to neutron autoradiography, we established that 30 μm is the thinnest nominal thickness that can be used for conventional neutron autoradiography without making thickness corrections [31,48]. On the other hand, the present analysis of the biological imprint formation showed that the sharpness of the imprint image increases as the sample gets thinner. As mentioned before, the imprint is formed by the photodegradation of the detector, which is more severe in the regions where the tissue is thinner and therefore less protective. Therefore, the imprint will be less pronounced for thicker samples. This fact is illustrated in the kidney autoradiographic images of Figure 10. The characteristic tubules of the renal cortex observed in Figure 10a are clearly revealed in the imprint presented in Figure 10b. The imprint obtained from the 10 μm section faithfully reproduces the histology of the sample, making the cell nucleus distinguishable. On the contrary, in the example corresponding to 30 μm thickness, only the glomerulus is well defined, both in the histology and in the imprint. The examples also show the fading effect in the 10 μm samples (Figure 10c), where fewer and smaller nuclear tracks are obtained than for the 30 μm sections (Figure 10f). This observation is consistent with the quantitative results presented in Figure 10b. The choice of tissue thickness involves a compromise between fading of nuclear tracks and imprint quality. While thicker sections prevent fading, thinner sections produce clearer imprints. As for boron microdistribution, these examples of normal kidney coming from BDIX rats infused with BPA do not show a preferential accumulation in any of the observed structures (not in tubules or in glomeruli).

The degree of detail that the imprint is required to reproduce will depend on the type of tissue and on the resolution at which the boron microdistribution needs to be studied. For instance, we had already noted that lung sections are more challenging to analyze than other tissues due to its physiological holes (alveoli, bronchi, and bronchioles) [31]. When acquiring photographs for track quantification, the smaller holes may go unnoticed since it is not possible to determine the origin of the blank regions in an autoradiographic image without imprint. In fact, they could be the result of the absence of tissue in that area or the result of the stochastic character of the neutron capture reaction in boron [31]. In this case, the use of tissue sections of 30 μm nominal thickness would be adequate to overcome the physiological gaps as the imprint contour clearly outlines the holes present in the histology. However, if the aim were to study boron microdistribution in the different structures of the tissue, it would be necessary to generate a more detailed imprint from a thinner section. The tissue thickness can be chosen depending on the analysis requirements, thus providing versatility to the technique.

### 3.9. Evaluation of Staining Agents

**Figure 11:**
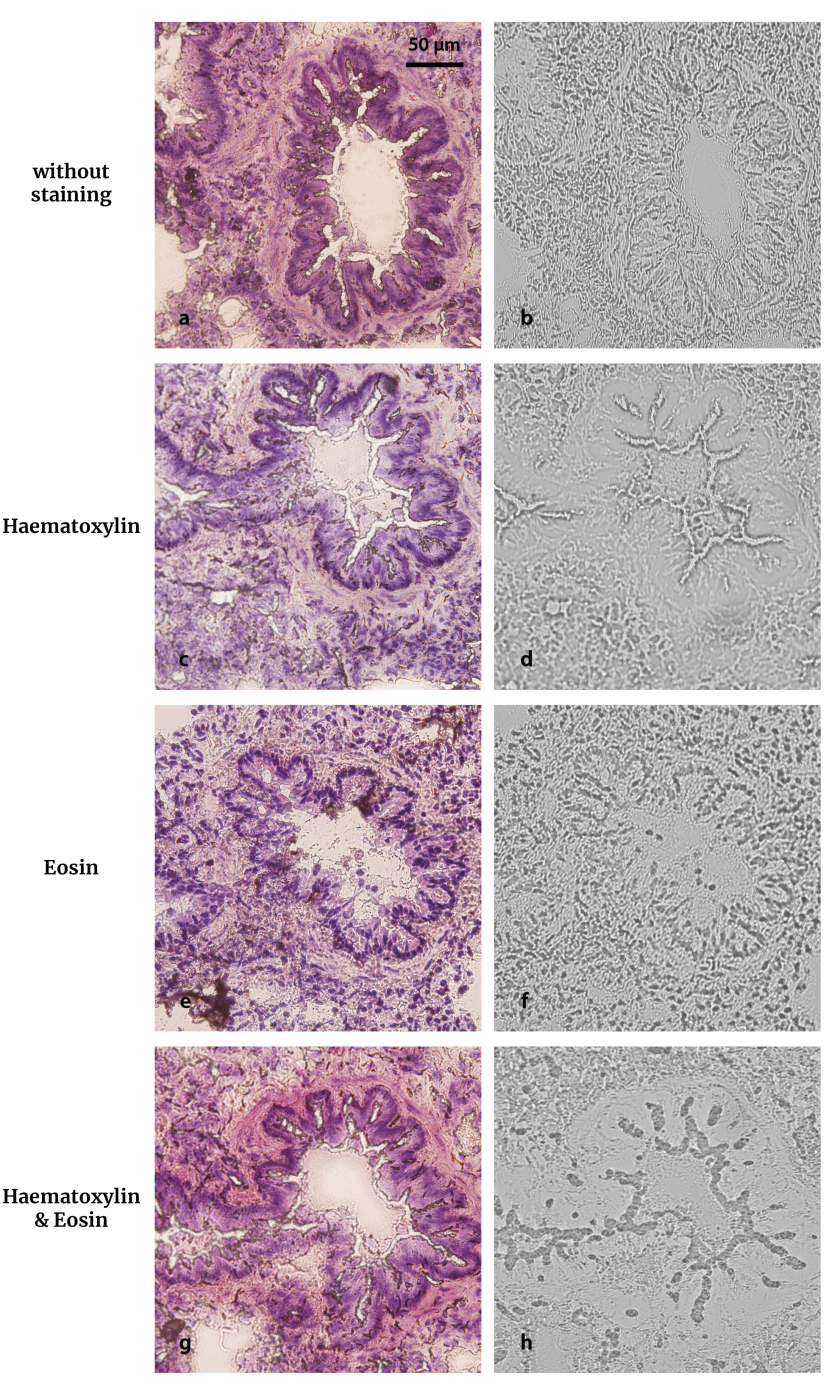
The effect of staining of tissue sections in the UV-C imprint. BDIX normal lung (3 h post GB-10 infusion, 50 mg ^10^B kg^−1^ bw) sliced to yield consecutive sections of 10 μm thickness (left) and their corresponding biological imprints in Lexan after a UV-C 10 min exposure (right) in different conditions: (**a,b**) no prior staining, (**c,d**) hematoxylin staining (15 min) prior UV-C exposure, (**e,f**) eosin staining (3 min) prior UV-C exposure, and (**g,h**) H&E staining prior UV-C exposure. In order to compare the histological images with the imprints, all sections were (re)stained with H&E after UV-C exposure. The imprints were revealed with PEW for 4 min.

In order to reduce fading in thin sections, the possibility to include a staining step between neutron irradiation and UV-C exposure was explored. In a previous work on cell cultures, we demonstrated that staining the cells (cultured on Lexan foils and incubated with BPA) with hematoxylin prior to UV-C exposure yielded very clear imprints and substantially reduced fading [35]. Based on this experience, different staining combinations were tested. The results of these assays are illustrated in Figure 11, where we explored the same ROI in consecutive sections coming from a BDIX normal lung sample. The staining conditions were hematoxylin (H), eosin (E), or a combination of them (H&E) prior to UV-C exposure (10 min). The epithelium contour of the bronchiole is clearly reproduced in the imprint without the need of prior staining (Figure 11b). While E staining allows the identification of cell nuclei in the corresponding imprint (Figure 11f), both H and H&E produce an excessive shift among the regions of higher staining intensity that mask the structures of interest (Figure 11d,h). Indeed, the smooth muscle cannot be distinguished from the epithelium. From the presented results, it seems that no staining would be required to obtain contours of the tissue structures, while eosin would be useful if higher resolution is desired. Nevertheless, specific applications (e.g., boron accumulation in vascular tissue) could require selective staining to highlight the desired structure in the imprint.

### 3.10. Final Comparison between PC and PADC

**Figure 12:**
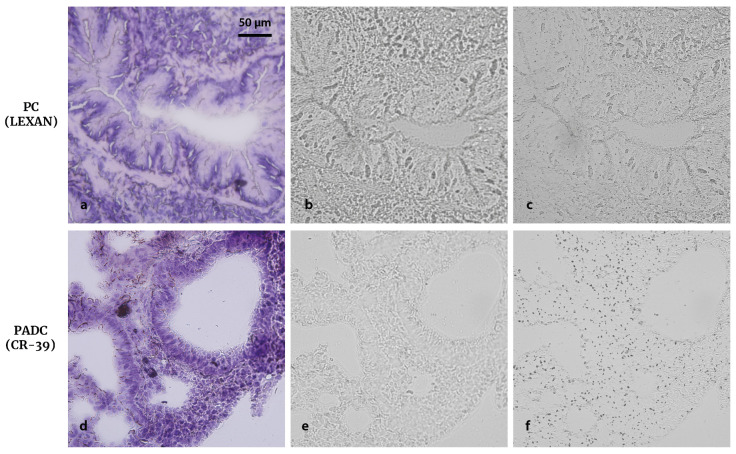
Comparison between Lexan and CR-39 in terms of observation of imprint and nuclear tracks. Histological images of BDIX normal lung (3 h post GB-10 infusion, 50 mg ^10^B kg^−1^ bw) sliced to yield sections of 10 μm thickness (**a,d**) and their corresponding imprints (**b,e**) and nuclear tracks (**c,f**) in Lexan (above) and CR-39 (below). Neutron fluence: 10^12^ n cm^−2^. The samples were stained with eosin prior UV-C irradiation. UV-C exposure time: 5 min for Lexan and 6 h for CR-39. Etching time: 4 min with KOH for Lexan and 32 min 35 s with NaOH for CR-39. Original magnification: 40x.

As a final comparison, consecutive samples were mounted both on PC-Lexan and PADC-CR-39 and processed at the optimal conditions determined for each NTD. The sections were stained with eosin prior UV-C exposure. While well-defined imprints of samples mounted on PC were obtained with only 5 min UV-C exposure, irradiations of about 6 h were necessary to yield imprints on PADC, regardless of previous staining. However, PADC NTDs posed the advantage that they did not exhibit the fading of nuclear tracks already seen for PC. This comparison is illustrated in Figure 12, where the histology, imprint, and nuclear track images are shown for lung sections from normal BDIX rats infused with GB-10. It must be taken into account that track density cannot be compared between detectors since they have different detection thresholds [41]. PADC has a higher sensitivity as nuclear track detector than PC but is more resistant to UV-C photodegradation. From a qualitative inspection of the autoradiographic images, boron seems to accumulate homogeneously. Nevertheless, in order to confirm this observation, a comprehensive quantitative analysis should be performed. Moreover, as we previously stated in Section 2.2, protons from the NNC reaction could be also revealed in the etching process. Other groups have reported the possibility to desensitize CR-39 through an etching with KOH solutions [21,43]. However, this process could affect the imprint quality, so its convenience should be evaluated.

In general, the following steps should be applied to generate imprints in Lexan:

1. Biodistribution study at the conditions established in the animal mode;
2. Tissue sectioning at 10 μm, unless a specific thickness is stated;
3. Neutron irradiation at 10 ${}^{12}$ n cm ${}^{-2}$;
4. Eosin staining, unless a specific agent is required;
5. UV-C exposure;
6. Staining for observation;
7. ROI determination;
8. Etching with PEW for 4 min;
9. Re-exploration of ROIs;
10. Nuclear track density determination;
11. Application of a correction factor related to fading;
12. Boron concentration determination.

Depending on the biological matrix to be studied and the resolution requirements, the conditions for applying this novel technique could vary. UV-C absorption might be different for each tissue and selective staining agents could highlight structures of interest not only in the histology but also in the imprint. The use of PC will require the application of a correction factor to consider fading in thin sections. Further work will be devoted to determining these factors for the tissues of interest. When only a contour of the tissue was needed to improve co-registration of histology and autoradiography, the use of PADC could be useful. Further work will be devoted to applying the neutron autoradiography technique with UV-C sensitization for the quantitative analysis of boron distribution in animal models of interest in the context of BNCT. For accurate track density analysis in these images, advanced image analysis tools as segmentation algorithms based on machine learning should be explored.

## 4. Conclusions

In this work, we extended neutron autoradiography combined with UV-C sensitization to tissue sections. We were able to generate tissue imprints that reproduce histological characteristics. We have revisited the parameters related to both sample preparation and NTD processing in order to establish optimal conditions for this biological matrix. An exhaustive analysis of these parameters allowed us to explore different biological models of interest in the frame of BNCT. In order to quantify boron microdistribution, further work will be devoted to adapting the technique to the specific requirements of the animal models under study.

## Figures and Tables

**Table 1:** Relative track density (RTD) for liver samples (30 μm) mounted on Lexan, irradiated with 10^12^ n cm^−2^ for three different conditions: with no UV-C exposure (n), exposed to UV-C for 10 min before neutron irradiation (UV-C+n), and exposed to UV-C for 10 min after neutron irradiation (n+UV-C). For each condition, a total of 2 samples and 50 images per sample were analyzed. Etching time: 4 min. The calculated boron concentrations for each sample are also presented. Results are reported as the mean value ± 1 SE.

Condition	n	UV+n	n+UV
RTD [%]	100 ± 16	92 ± 15	91 ± 12
^10^B cc [ug g^−1^]	7.3 ± 0.7	6.6 ± 0.8	6.5 ± 0.6

## Data Availability

Data available on request from the authors.

## Abbreviations

The following abbreviations are used in this manuscript:

BNC	Boron neutron capture reaction
BNCT	Boron neutron capture therapy
bw	Body weight
BPA	Boronophenylalanine
CEv	Evaporation coefficient
GB-10	Decahydrodecaborate
H	Hematoxylin
E	Eosin
NTD	Nuclear track detector
PADC	Polyallyldiglycolcarbonate
P	Parenchyma: neoplastic cells
PC	Polycarbonate
PEW	Etching solution prepared with KOH, water, and alcohol
ROI	Region of interest
RTD	Relative track density
SEM	Scanning electron microscope
S	Stroma: non-neoplastic connective tissue
UV	Ultraviolet
Vb	Bulk velocity
